# The Pathology of Fevers

**Published:** 1855-01

**Authors:** N. S. Davis

**Affiliations:** Prof. of Pathology, Practice, and Clinical Medicine in Rush Med. College; member of the Amer. Med. Association, &c. &c.


THE NORTH-WESTERN

MEDICAL AND SURGICAL JOURNAL.

NEW SERIES.

VOL. IV. JANUARY, 1855. NO. 1.

### ORIGINAL COMMUNICATIONS.

ART. I.—*The Pathology of Fevers; or observations critical and experimental in reference to the nature, varieties, causes and treatment of Fevers.*—By N. S. DAVIS, M. D., Prof. of Pathology, Practice, and Clinical Medicine in Rush Med. College; member of the Amer. Med. Association, &c. &c.

### CAUSES OF FEVER.

HAVING in former articles considered somewhat in detail, the nature and varieties of Fever *, I shall now proceed to examine briefly those agencies or causes which are capable of so acting on the human system as to give rise to febrile phenomena. In the articles just alluded to, I have claimed that all the tissues of the living body were possessed of two essential and elementary properties. By the first, the elementary cells and molecules are disposed to assume and maintain a certain definite position in relation to each other, constituting the organization or texture of parts.—This definite arrangement of the matter composing the several elementary structures, I have called their *tonicity*. Thus when the affinity of cell for cell &c., is diminished in any or all the tissues, rendering them lax in texture and consequently feeble in the performance of their function, the *tonicity* is said to be diminished; and *vice versa*. By the second, each organized cell, fibre, or tissue is rendered capable of receiving and responding to impres-

* See a series of articles published in the 2nd vol. of the North Western Med. & Surg. Journal.

sions. By it, the nerves and fibres are rendered capable of receiving and transmitting impressions; the muscular fibre, of contracting on the application of nerve influence, electricity or other stimulants; the capillaries, of responding to the presence of blood in them, &c. &c. This property, this inherent in all living matter, I have called *susceptibility;* and have considered it capable of being increased or diminished by the action of various agents.

So long as these two properties, tonicity and susceptibility, preserve their natural condition and relations to each other, so long will the various functions of the system remain normal and health be preserved. If through any morbific influence one or both of these properties are disturbed in only one organ or tissue, *local* disease or derangement of function will follow. If the morbific influence, acting through the blood, reaches the properties of all the tissues at the same time, universal disturbance of function must follow.

Hence I have defined *fever* to consist essentially in a disturbance of the elementary properties of the whole body; while the particular character of the fever will depend on the *direction* given to the altered properties. If the cause act in such a manner as to increase tonicity and susceptibility throughout the tissues, a true stenic or inflammatory grade of fever will be produced; if in such a way as to diminish or depress both properties, the resulting fever will necessarily exhibit a low or *typhoid* character; and if the cause possesses specific qualities capable of depressing one property and at the same time exalting the other, it will induce a fever of a specific character, differing alike from the inflammatory on one side and the typhoid on the other. And here I have claimed an explanation both of the *oneness* or unity of fevers, and their diversity. All fevers are a *unit*, inasmuch as they arise from disturbance of the same general properties, while they are widely *diverse* in their phenomena and results, because these properties are disturbed in different directions by exciting causes differing essentially in their nature.

In accordance with these views concerning the nature of fevers, I shall consider the *causes* under the three following heads, viz.:

1st. All such agents as are capable of exalting or increasing both susceptibility and tonicity.

2nd. Such as directly diminish both these properties.

3d. Such as exert a specific influence, increasing susceptibility and either diminishing tonicity or allowing it to remain normal.

Under the first of these divisions are included all causes capable of generating the fevers termed *Synocha*, by Cullen, Irritative and Inflammatory by Tweede, and Irritative or simple continued fevers by Dr. Wood. They are not numerous, nor are the fevers to which they give rise, as frequently met with as the other varieties, unless we include under this head those symptomatic fevers depending directly on local inflammation. The latter is so frequently the cause of this grade of fever, that many have expressed the opinion that it is always accompanied by inflammation in some of the structures of the system. Though not intending to include a consideration of mere symptomatic fever in these articles, yet an inquiry into the mode by which general febrile symptoms are produced as a consequence of local inflammation, may not be wholly irrelevant or unprofitable. That all acute inflammations of any considerable extent are soon followed by those symptoms which are universally denominated febrile, is well known; but *in what way* does the local affection act en the general properties to such an extent as to produce that universal derangement of functions which constitutes fever?

A satisfactory answer to this question is not as easily given as many would suppose. Broussais attributes the general disturbance to an irritant impression transmitted, through the medium of the nerves, from the inflamed part to the Brain, and from this reflected over the whole system. If this were true, we should always be able to trace special cerebral disturbance anterior to the general febrile phenomena, and the latter should bear a direct ratio to the former; neither of which is in acccordance with the daily observations of the profession

Others have attributed the general symptoms to the *shock* produced by the rapid congestion and other changes which take place in the inflamed part. But the word *shock* is as mysterious as any other, and consequently explains nothing. A larger number of writers, seeing that the fibrine of the blood is rapidly increased in most inflammations, have attributed the febrile excitement to the stimulant effects of the superabundant quantity of this constituent. Two facts, however, are strongly opposed to this view. The first is that the febrile symptoms often appear in their greatest intensity before the accumulation of fibrin has taken place to any considerable extent in the mass of the blood, and still more frequently disappears before the excess of fibrin is removed. The second fact is that the intensity of febrile action does not bear any uniform and direct ratio to the degree of increase in the quantity of fibrin. These may not be sufficient to justify the conclusion that the excess of fibrin exerts no exciting or irritating influence, but they are sufficient to show that it is not the *chief* cause of such action.

I am induced to believe that local inflammation induces fever either by its effects on the nervous system, or by its interference with some of the excretory functions; and in most cases by both these combined. The effect of inflammation on the nervous system alone is seldom capable of inducing more than a very moderate and ephemeral form of fever. Yet we see very often patients suffering under a distinct general febrile movement, resulting solely from a *whitlow* on the finger, for instance. The part involved in the inflammation is not such as to interfere with any secretory or eliminating function, neither of sufficient extent to materially alter the proportion of fibrin or any other constituent of the blood. But the extreme pain resulting from the irritation of nervous filaments involved in the part, is of itself sufficient to disturb, in a moderate degree, all the functions of the system; causing rigors, pains in the head and back, increased heat of skin, and frequency of pulse, restlessness, &c. Doubtless in such cases sufficient morbid impression or irritation is transmitted through the nervous filaments and connections to produce the general disturbance.

The suddenly developed paroxysms of fever which result from the irritation of worms in the alimentary canal arise chiefly from the same cause. The portion of mucous membrane in contact with the worms, is not inflamed to such a degree as would either suspend secretion or fill the blood with fibrin; but it involves a condition of the nervous filaments in the part, which is rapidly transmitted to the ganglionic and cerebro-spinal systems; thereby inducing rapidly a general excitement, which is usually as ephemeral or transitory as it is rapidly developed.

But in all the more important and lasting cases of symptomatic fever, the structures involved in inflammation are such that disturbance of one or more functions of importance necessarily results co-incidently with the beginning of the local disease. And such is the intimate relation and dependence of one important function on all the others, that no sudden disturbance of one can take place without inducing more or less change in all. Thus disease, occupying any considerable part of the cutaneous surface, suspends the elimination from the skin, causing, on the one hand, rapid accumulation of perspirable matter in the blood; thereby exerting a universal irritant influence; and on the other, equally rapid accumulation of heat from the diminished insensible evaporation, by which, in health, the caloric on the surface of the body is constantly being rendered *latent*, and conveyed away. From these two alterations—the retention of irritating matter in the circulating fluids, and the accumulation of caloric, general febrile symptoms are very quickly developed. Again, inflammation of any portion of the respiratory apparatus sufficient to materially impede respiration quickly alters the interchange of oxygen and carbonic acid through the lungs, thereby altering the quality of the blood, and, through it, the functions and properties of the system.

If we bear in mind the fact, that every change in the capillary circulation is accompanied by a change of function, we shall see that the first step in the inflammatory process, namely, determination of blood to, and its accumulation in the inflamed part, almost necessarily involves more or less alteration in the capillary circulation generally, and consequently must induce at once some general disturbance.

Add to this the irritant impression made on the nerves of the part, and through them transmitted to the important nervous centres, together with the rapidly increasing alterations of the blood, by the retention of materials designed for elimination; and there can be no difficulty in comprehending the process by which active local inflammation is soon followed by that general functional disturbance which constitutes fever.

But the same *sthenic* or inflammatory grade of febrile action may, and often does occur from other causes, without the intervention of any local inflammation.

The most important of these causes is the application of cold to the surface of the body, and the rapid alternations of temperature. To operate as an exciting cause of the sthenic grade of fever, the application of the cold must be made suddenly, and continued only for a limited period of time. Cold applied in this way produces a twofold effect—namely, a direct diminution of the capillary circulation throughout the exterior of the body, with corresponding diminution of the cutaneous exhalation, and of organic or molecular action. While this condition exists there is not only a failure to eliminate from the blood effete matter, in the form of insensible perspiration, but the restricted organic action in the exterior retards, of course, the process of nutrition, disintegration, and calorification. Hence, when the action of the cold ceases, the blood contains the retained effete perspirable matter, the exterior tissues contain particles that should have been removed by the process of disintegration, which prove directly irritant to the nervous and vascular systems. All these combined tend to develop rapidly an *irritant* influence throughout the whole system. This irritant action, coupled with the accumulation of caloric from the diminished cutaneous exhalation, soon establishes the re-action or general febrile movement. If the action of the cold has been pretty intense, but temporary, the resulting febrile action will be characterized by increased nervous sensibility and high vascular excitement. But if the action of the cold is less intense and more protracted, the sensibility of the tissues becomes diminished instead of increased; and though fever may result from the ultimate accumulation of excrementitious matter, yet the grade of morbid act on will be *asthenic* rather than inflammatory.

The action of cold as an exciting cause of fever is much increased if it is accompanied by dampness. An atmosphere highly saturated with moisture, not only increases the effects of the cold on the cutaneous function, but it diminishes to a great extent also the exhalation of aqueous vapor from the lungs, thereby causing the retention of a small amount of animal matter, and a large amount of caloric which should have been rendered latent in the aqueous vapor thrown out with each expiration. Hence it is that all writers agree in attributing common continued and ephemeral fevers, characterized by sthenic excitement, to exposure to cold and wet, and sudden atmospheric changes. Thus Dr. Wood says, "the most frequent cause is probably exposure to cold. Persons who have been caught in a cold rain, and remain for some time with their wet clothes upon them, are apt to be attacked." And he very properly adds that, "whatever is capable of producing *irritation* in one or more of the important organs of the body may give rise to this kind of fever." Hence, the presence of indigestible food in the stomach, of worms in the bowels, and the irritation of teething, are generally enumerated among the exciting causes of irritative fever. But all the causes which have been mentioned except local inflammation, so frequently exist without producing fever, that most writers acknowledge the necessity of some previous predisposition. In what that predisposition consists few have attempted to explain.

It will doubtless be found in most cases, to depend on an impairment of the mutual sympathy and compensating action which always exists, in a strictly healthy condition of the system, between the more important functions of the body. This sympathy is most prominently displayed between the skin and kidneys on the one hand, and the lungs and liver on the other.

The two first eliminate from the system chiefly nitrogenous matter, and the two last carbonaceous. The compensating action of the two first is so frequently and prominently exhibited that the most careless observer would scarcely fail to notice it. Thus whenever a healthy individual is exposed during the day to a moderately cold atmosphere, the exhalation from the surface is much diminished, while the quantity of urine discharged is proportionately increased; and so long as this continues no derangement of health ensues from the action of the cold. But let this compensating action of the kidneys cease, and the action of the cold on the surface, quickly produces the retention of a sufficient quantity of efiete and waste matter in the blood to arouse the susceptibility of the whole system and establish the phenomena of fever. There are many facts which induce us to believe that a similar functional relationship exists between the lungs and liver, although the relative functional activity of both is less open to observation or manif st to the senses. Thus the occurrence of a bilious diarrhœa frequently relieves very much the consequences of severe obstructions in the lungs, and occasionally proves critical, being followed by speedy convalesence. Again, that season of the year and those locations which give rise to an atmosphere both warm and damp, are accompanied by the smallest degree of elimination of carbonic acid gas from the lungs, on the one hand, and the prevalence of diseases characterized by an excessive flow of bile on the other. This is verified during the latter part of summer and autumn in all malarious districts; while just the reverse is seen during the cold season when the atmosphere is dense and more dry.

Nature has thus provided the human body with organs capable of affording to each other a mutually compensating action, at least to an extent sufficient to preserve it from harm from the ordinary changes in the elements around us.

But whenever that mutual sympathy is interrupted or materially impaired by previous exposures or debilitating influences, then it is that the action of any cause, capable of disturbing one or more of the important functions of the body, is sufficient to develope febrile phenomena. And it is more than probable that the *predisposition* to this or that disease, of which authors so frequently speak consists in this impaired power of compensating action between the different excretory organs of the system.

Prominent among the predisposing causes of the sthenic grade of fever, are age, temperament, and season.

It is during the period of childhood and youth, before the physical system has attained its full developement, that far the larger proportion of cases of this form of disease occur. It is during this period of life that the blood contains the largest relative proportion of nutrient matter, the tissues their highest degree of susceptibility, and the organic movements take place with the greatest rapidity. Hence it is the period above all others, when excited action is most readily induced. The strongly marked sanguin temperament presented in adult life doubtless predisposes to the same kind of morbid action. The essential elements of such a temperament are a fully developed vascular system, a ready excitability, and blood containing a full proportion of red corpuscles; all conditions favorable to the occurrence of excited action coupled with strength. The influence of seasons in predisposing to the prevalence of the common irritative or sthenic cases of fever is perhaps not as readily observed as that of age. My own observations, however, have led me to the conclusion that they are met with most frequently at the beginning and the end of the cold season.

The steady and intense cold of mid winter generally depresses the susceptibility of the system too much, and the heat of summer induces too great laxity or loss of tonicity, while the moderate coldness of early spring and late autumn gives the proper degree of tonicity without depressing the susceptibility, thereby presenting the system in a condition favorable to the morbid influence of the frequent changes which characterize these seasons.

*Causes which depress the elementary properties.*

The second class of causes which require consideration, though numerous and diverse in their nature, yet all agree in two essential qualities, namely, the power to act upon or influence the general or elementary properties of all the issues, and to act in such a manner as to depress or diminish those properties. This latter quality distinguishes them from the agencies mentioned in the first class.

Most, if not all of the causes included in this class also differ from those mentioned in the first in another respect.

The latter, as we have seen, act primarily on particular organs and functions, and through them on the general properties of all Hence the general functional disturbance or febrile movement, resulting from their action, is a secondary effect; while those belonging to the class now under consideration are capable of exerting a direct influence on those properties which belong to all the tissues, and consequently capable of inducing, primarily, alterations of function in all the organs of the body. The chief causes included in this class are the following, viz.: The protracted influence of cold; deficient nutriment; excessive exercise; continued mental depression and anxiety; impure air; and some of the products of the decomposition of animal and vegetable matter.*

These several causes may act singly, though two or more of them frequently co-exist and exert their influence on the sytem at the same time. Thus protracted cold is often united with excessive exercise or insufficient food. So too, among the poor especially, impure air is often associated with both mental depression and unwholesome food. Indeed, it is rare that any one of the causes named, except the animal poisons, act with sufficient intensity to cause the direct developement of idiopathic fever. And yet they are each of sufficient importance to require a brief examination in reference to their *modus operandi.*

*Cold.* "Under the local influence of cold," says Dr. Wood, "the tissues shrink, the capillaries circulate the blood less vigorously, secretion is checked, sensibility is impaired, and the part becomes pale from the want of blood, or purplish from its stagnation. Should the application continue with intensity, a deadly whiteness ensues, circulation ceases entirely, sensibility is lost, and, though vital power is not immediately destroyed with the loss of action, local death soon follows unless the cause be removed.

When the influence is general, the whole surface undergoes the change above described; while the interior functions exhibit a similar depression. The heart acts by degrees more and more feebly a general torpor steals over the senses, the movements become tottering from muscular weakness, and the benumbing influence extends at last to the brain, giving rise to wandering of the thoughts, drowsiness, and finally an irresistible propensity to sleep."†

Dr. Williams in his Principles of Medicine remarks as follows, viz.: "Cold, on the other hand, is directly sedative. It contracts

* Under this head I include what is generally called contagion.

†See Wood's Practice of Medicine, vol. 1. p. 142.

tissues and vessels, especially the arteries, and thus at first renders parts pale and shrunk. In persons of feeble circulation, after bathing, the fingers are sometimes quite bloodless and numb from this cause; the cold having quite closed up the arteries. But cold also retards the passage of the blood in the capillaries; the viscidity of the Liquor Sanguinis seems to be increased; globules stick to the sides or move but slowly, and the part soon becomes purple or blue, from the congestion of blood in it. There is also much internal congestion from the intropulsive operation of the cold—that is, the external parts being constricted and obstructed, blood accumulates more in internal parts, and the heart's force is more expended on these." The idea here expressed, that the action of cold produces "internal congestion," from its *intropulsive* operation, is one very generally entertained. But Dr. Wood goes further, and not only claims internal congestion of blood, but of nervous energy also. His language is as follows: "Another method in which cold may produce disease is, by the concentration of blood and of nervous energy in the interior organs, consequent upon their retrocession from the skin, and the parts which sympathize with the skin. It is obvious that the quantity of blood is diminished in the superficial vessels. It must necessarily, therefore, be increased in those within. It is equally certain that nervous action is depressed upon the surface; and it is highly probable that this also is concentrated in the interior organs. Excess of blood, and excess of nervous energy combined, cannot but frequently eventuate in disease." This language plainly implies that nervous energy is a something not only capable of circulation, but definite in *quantity*, like the blood; and like it, when driven from one set of organs, accumulating in another. He thus gives to the mere *property* of a particular tissue, all the essential qualities of a separate *entity*, as plainly as Van Helmont did to his *Archeus*. It is scarcely necessary to say that I regard such an assumption as purely hypothetical, and better calculated to mislead than to enlighten the reader. But, aside from this, is there anything in the phenomena which accompany exposure to cold, which really indicate the existence of either an *increased* amount of blood, or of nervous energy in the internal organs of the body? Certainly there is not. so far as those phenomena are deta led to us by Drs. Wood, Williams and others who have written on the subject; or by those who have suffered in their own persons the effects of extreme cold. To study this subject intelligibly we must keep in mind the well known phenomena of congestion or accumulation of blood in the internal organs, that we may the more accurately compare such phenomena with those represented to arise from the effects of cold. By internal organs we usually meant those viscera which occupy the cavities of the cranium, the thorax and the abdomen.

If the reader will turn to the accounts of congestion in the Brain, the Lungs, Heart, or abdominal viscera, given by any of the writers on Pathology or Practical medicine, he will readily perceive striking differences between the sympt ms there detailed, and those resulting from the action of cold.

Simple congestion or accumulation of blood in the brain, for example, produces a sense of fulness, heaviness and vertigo; and if it be increased, these give place to stupor, coma. or paralysis, with fulness of the pulse and more or less laborious respiration. If *increased nervous energy* or irritation exist in the brain at the same time, we have in addition to the other symptoms, increased heat of the head, flushed face, fulness of the carotids, and delirium or spasmodic muscular action, or both. In searching the interesting accounts of the action of extreme cold, given us by Sir Joseph Banks, Dr Solander, Dr. Edward Daniel Clarke, and others, we shall find no mention of any of these symptoms. On the contrary the only appreciable change in the brain is a gradual impairment and final suspension of its action. Hence the first morbid feeling is that of want of power, the acts of the will are performed with difficulty; the individual feels conscious that thinking requires an effort, and there is nothing he so much desires as to suspend all thought, all effort of mind or body, and be allowed to sink, not into coma, convulsions, or paralysis, but a perfectly quiet sleep; during which the already feeble action should cease altogether. So different are these symptoms from those arising from accumulation of blood and nervous energy in the brain that Dr. John Whiting very justly observes, "that it is hardly possible to determine whether they are the direct result of the action of the cold as a sedative on the brain, or whether they depend on *a want of the due supply of blood to that organ*, on account of the diminished action of the heart." It is true that Dr. Kellie has reported in the Edinburgh Medical Journal some cases of death after severe exposure to cold, in which he found more or less serous effusion in the brain; but in all these, death took place, not from the *direct* action of the cold, but sometime after both "heat and circulation had been restored."

If we examine the symptoms derived from the heart and lungs, we shall find still less real evidence of congestion or accumulated nervous energy during the action of the cold.

Pulmonary congestion is generally accompanied by two things manifested in a greater or less degree: first, dyspnœa, or at least imperfect inspiration, and imperfect decarbonization of the blood. But difficulty of breathing, or imperfect respiration, is nowhere mentioned as one of the phenomena observed during extreme cold, and so far from there being evidence of deficient decarbonization of the blood, various experiments go to show that blood remains arterial through out the whole vascular system.

Thus Chossat found in animals, killed by cold, the blood of arterial color in both ventricles of the heart; and Sir Astley Cooper found that "on plunging kittens into ice cold water the arterial blood did not become venous in the veins."

In regard to the heart, Dr. Wood, himself says, that under the influence of cold it "acts by degrees more and more *feebly*;" which surely is not an evidence of the "accumulation of blood and nervous energy" in it. The latter would necessarily lead to increased action in the heart, calculated to expel the supposed increased quantity of the former; thereby exhibiting an unusual degree of cardiac impulse, instead of one progressingly more and more feeble. A writer in the Encyclopædia of Practical Medicine comes much nearer the truth when he says, "it is very probable that the sedative action of cold on the circulating system is to be ascribed to a *diminution* of the *irritability* of the muscular fibres of the heart and blood-vessels." Among the many cases recorded of death from the direct effects of cold, I have searched in vain for any evidence of internal congestions, derived from *post mortem* examinations. Indeed, from a careful examination of the subject, I have been led to conclusions entirely different from those commonly entertained and very fairly expressed in the quotations from Drs. Williams and Wood. The common opinion is based directly on the assumption that the quantity of blood is diminished in the surface and parts most exposed to the cold, and therefore must "*necessarily*" be *increased* in the parts within. The idea of "*intropulsive action*" is founded altogether on the supposition that the blood forsakes the surface—that its quantity is absolutely diminished in the exterior parts. But is this fundamental or primary proposition true? It is true that the application of cold condenses the tissues, thereby causing the vessels to be smaller, but it equally condenses the blood and fluids in them, rendering, as Dr. Williams says, the liquor sangwinis more viscid, causing the globules to "stick to the sides of the vessels or move but slowly," and consequently directly retarding the passage of the blood in the capillaries. At the same time that the blood is thus altered in its density and movement through the capillaries, the susceptibility of the tissues is diminished, and those organic movements by which new particles are added to the elementary structures and old ones removed, are retarded or entirely suspended.

This, of course, retards in an equal ratio the secretion, the production of animal heat, and the change from arterial to venous blood in the systemic capillaries. The inevitable consequence of all these changes would be, not an "*intropulsive movement*," by which the blood and nervous energy should accumulate within, but a direct and progressively increasing retardation of both circulation and organic change in the exterior or parts exposed to the cold; thereby allowing absolutely less blood to be returned to the heart in a given time; and what is returned has a diminished temperature, and less of the materials usually derived from the systemic capillaries—in other words, less venous in its color. The blood returned to the heart thus reduced in its temperature and quantity, fails to sustain the susceptibility and action of the heart, brain, &c.; thereby causing that *sedative* action, which, by long continuance or increase, results in entire suspension of action and death.

Such, I believe, is the true mode in which cold produces its effects on the human system. It condenses the blood and tissues, and diminishes the elementary susceptibility; thereby retarding organic and capillary action, the production of animal heat, and secretion, in the parts exposed; and causing the blood to return to the heart in less quantity, and at a lower temperature, by which the functions of the brain, the heart, and all the internal organs are enfeebled or suspended, according to the intensity and duration of the cold.

If the application of cold be of short duration and of only a moderate degree of intensity, the temporary condensation and diminished action only causes the retention in the part of sufficient blood and effete matter to prove gently irritant or excitant as the action of the cold ceases, and the tissues again relax. No sooner, however, has the cold ceased than this moderately irritant action induces an increased circulation and organic action, and thereby increased het and redness, and this constitutes the *reaction* from the effects of cold When the re-action thus induced is moderate, it soon disappears and the sensation of warmth, which accompanies it, is pleasant rather than otherwise. In persons with *lax* tissues and morbid susceptibility, these slight reactions from the temporary application of cold, repeated at suitable intervals, may be made to act as an efficient tonic. But if the application of cold be more protracted or intense, the altered density together with the stasis of blood and fluids, so alters and excites the tissues on the return of warmth, that the re-action constitutes inflammation. The latter may end in the formation of *chilblains*, in suppuration, or in gangrene.

It is the long continued influence of a moderate degree of cold which induces results of the most etiological importance. In such cases, the degree of cold is not sufficient to induce rapid changes, but its long continuance gradually lowers down the susceptibility of the tissues, and sooner or later, accumulates in them sufficient effete matter from the diminution of organic action and secretion to disturb the various functions of the body. Such disturbance, however, will always be of an asthenic or depressed character.

The *poor*, during the cold season of the year, not unfrequently suffer from this protracted influence of moderate cold, often determining in them the developement of fevers of a low grade.

Hence, Dr. Wood very justly remarks, that, "the long continued influence of a moderate degree of cold is apt to wear out the powers of reaction, especially when aided by want of food, and other sedative agencies A state of the system is thus produced, highly favorable to typhous and scorbutic diseases; and when the individual may be attacked with another disease, even though inflammatory, it is apt to assume a low asthenic, or typhoid character.

I believe, also, that a strong predisposition may thus be developed to phthisis or scrofula, or an existing pre-disposition to these affections called into action."

It is not probable, that, under ordinary circumstances, the influence of the cold in depressing the properties and functions of the system, is of itself sufficient to give rise to the developement of fevers of an asthenic grade; in other words, it is not often a direct *exciting* cause; but acts rather as one of the most important *predisposing* influences. By retarding the organic changes and depressing the properties, it *determines the grade* of fever, on the occurrence of another exciting cause, without being sufficient of itself to develope the more active morbid phenomena. Thus, most of the wide-spread and fatal epidemics of *typhus*, have prevailed in the winter; and many of them in the midst of extraordinary cold ones.

It is during the same season of the year also, that this disease is apt to occur, and prevail with severity, in military camps, where large numbers are but imperfectly sheltered from the cold for several successive weeks. Under all circumstances, the injurious effects are greatly increased by the co-incidence of sufficient nourishment. This is not owing solely to the deficiency of nutritious material in the blood from the want of a proper supply, but partly also, to the fact that active digestion directly increases the production of animal heat in a noted degree. This is easily demonstrated by direct experiment, as shown by me in an essay read to the American Medical Association during its annual meeting in May, 1851.

